# Prescriptions

**Published:** 1874-11

**Authors:** M. S. Bidwell


PRESCRIPTIONS—*By M. S. Bidwell.*— * * * Briefly to recapitulate :

(1.) A prescription is a confidential letter from a physician to a pharmacist, the latter having the right of custody, but not the right to make it public. The patient, being an interested party, has a right to a copy.

(2.) The druggist's business is to furnish whatever medicines the customer wants, whether prescribed by a doctor or not, the patient taking his own risk.

(3.) The physician, like any other scientific man, should be liberal in communicating what he originates, because most of his own knowledge is derived from others, and in this way only can science be advanced. But this obligation is ethical, not legal—something to be desired and recommended, not enforced.

I hope no part of the above will be understood to countenance the practice of "prescribing across the counter," or of prescribing by any other unqualified party. This is a nuisance in the drug business, and one that every intelligent and fair-minded pharmacist will endeavor to abate. * * *

